# Evolving Practice and Outcomes in Grade 2 Glioma: Real-World Data from a Multi-Institutional Registry

**DOI:** 10.3390/cancers16203514

**Published:** 2024-10-17

**Authors:** Lucy Gately, Katharine Drummond, Anthony Dowling, Iwan Bennett, Ronnie Freilich, Claire Phillips, Elizabeth Ahern, David Campbell, Megan Dumas, Robert Campbell, Rosemary Harrup, Grace Y. Kim, Simone Reeves, Ian M. Collins, Peter Gibbs

**Affiliations:** 1 Personalised Oncology Division, The Walter and Eliza Hall Institute of Medical Research, Parkville, VIC 3052, Australia; 2 Department of Medical Oncology, Alfred Health, Melbourne, VIC 3004, Australia; 3 Department of Neurosurgery, Royal Melbourne Hospital, Parkville, VIC 3052, Australia; 4 Department of Surgery, University of Melbourne, Parkville, VIC 3010, Australia; 5 Department of Medical Oncology, St Vincent’s Hospital Melbourne, Fitzroy, VIC 3065, Australia; 6 Department of Medicine, University of Melbourne, Melbourne, VIC 3010, Australia; 7 Department of Neurosurgery, The Alfred, Melbourne, VIC 3004, Australia; 8 Cabrini Hospital, Malvern, VIC 3144, Australia; 9 Department of Radiation Oncology, Peter MacCallum Cancer Centre, University of Melbourne, Melbourne, VIC 3010, Australia; 10 Sir Peter MacCallum Department of Oncology, University of Melbourne, Melbourne, VIC 3010, Australia; 11 Department of Medical Oncology, Monash Health, Clayton VIC 3168, Australia; 12 Department of Medical Oncology, University Hospital Geelong, Barwon Health, Geelong, VIC 3220, Australia; 13 Bendigo Health, Bendigo, VIC 3550, Australia; 14 Cancer & Blood Services, Royal Hobart Hospital, Hobart, TAS 7000, Australia; 15 Menzies Research Institute, University of Tasmania, Hobart, TAS 7005, Australia; 16 Ballarat Austin Radiation Oncology Centre, Ballarat, VIC 3350, Australia; 17 Department of Medical Oncology, South West Regional Cancer Centre, Geelong, VIC 3220, Australia

**Keywords:** glioma, IDH mutation, patterns of care, real-world data

## Abstract

**Background**: Grade-2 gliomas (G2-glioma) are uncommon. In 2016, RTOG9802 established the addition of chemotherapy after radiation (CRT) as a new standard of care for patients with high-risk G2-glioma, defined as subtotal resection or age ≥40 yrs. Here, we report current practices using real-world data. **Methods**: Patients diagnosed with G2-glioma from 1 January 2016 to 31 December 2022 were identified in BRAIN, a prospective clinical registry collecting data on patients with brain tumours. High- and low-risk were defined as per RTOG9802. Two time periods, January 2016–December 2019 (TP1) and January 2020–December 2022 (TP2), were defined. Survival was estimated using the Kaplan–Meier method. **Results**: 224 patients were identified. Overall, 38 (17%) were low-risk, with 35 (91%) observed without further treatment. A total of 186 (83%) were high-risk, with 96 (52%) observed, 63 (34%) receiving CRT, and 19 (10%) receiving radiation. Over time, CRT use increased (TP1 vs. TP2: 22% vs. 36%, *p* = 0.004), and the rate of biopsy (TP1 vs. TP2: 35% vs. 20%, *p* = 0.02) and radiotherapy alone (TP1 vs. TP2: 14% vs. 4%, *p* = 0.01) decreased. Median progression-free survival (PFS) was significantly longer in high-risk patients who received CRT (NR) over observation (39 months) (HR 0.49, *p* = 0.007). In high-risk patients who were observed, 59 (61%) were progression-free at 12 months and 10 (10%) at 5 years. OS data remains immature. **Conclusion**: Congruent with RTOG9802, real-world BRAIN data shows CRT is associated with improved PFS compared to observation in high-risk G2-glioma. Whilst CRT use has increased over time, observation after surgery remains the most common strategy, with some high-risk patients achieving clinically meaningful PFS. Validated biomarkers are urgently required to better inform patient management.

## Simple Summary

This study explored the current practices in the real world for grade 2 glioma, an uncommon primary brain cancer. Leveraging the prospective BRAIN registry, this study demonstrates that whilst sequential radiotherapy and chemotherapy improve progression-free survival in high-risk grade 2 glioma, the majority of patients are observed following surgery. In high-risk patients who are observed, 61% remain progression-free at 12 months, with 10% being progression-free at 5 years. This clinically meaningful progression-free survival suggests that validated biomarkers beyond the usual definition of high-risk are required to better inform patient management. Factors contributing to decision-making are underway.

## 1. Introduction

Grade 2 gliomas are relatively uncommon and account for approximately 10% of all primary brain tumours [1]. Historically, standard treatment included maximal surgical debulking followed by close observation, with radiotherapy generally reserved for use at recurrence or progression unless symptomatic. The notion of delaying radiotherapy arose in part due to the association with relatively high rates of cognitive decline [2] and the lack of survival benefit seen with upfront versus delayed radiotherapy [3], thus allowing toxicities to be deferred or even spared in a proportion of this patient population. 

However, in April 2016, seminal results were published by the Radiation Therapy Oncology Group (RTOG) demonstrating a significant survival advantage (13.3 vs. 7.8 years; hazard ratio for death, 0.59; *p* = 0.003) with the use of postoperative radiotherapy and multi-agent chemotherapy using procarbazine, carboplatin and vincristine (PCV) versus radiation alone following resection in patients who were deemed high-risk [4]. In this trial, high-risk was defined as either incomplete resection or age 40 years and older, regardless of the extent of resection [4]. The uptake of this new management strategy in the real-world population in Australia has not previously been reported.

This review aims to understand the current treatment patterns for grade 2 glioma in the first line and beyond, as well as the associated outcomes in a contemporary treatment setting. 

## 2. Materials and Methods

Data was extracted from the BRAIN registry [5] on 24 May 2024 for patients with grade 2 glioma diagnosed between 1 January 2016 and 31 December 2022, encompassing five metropolitan public hospitals, four metropolitan private hospitals, and three regional centres. The BRAIN registry captures comprehensive clinical data for consecutive patients diagnosed with a brain tumour at participating centres, including patient demographics, tumour characteristics, treatment, and outcome data. Extracted data included demographics, tumour histology and pertinent molecular features, treatment in first and subsequent lines, including the extent of resection (as estimated by the surgeon or post-operative imaging), receipt of radiotherapy and chemotherapy, and survival data. 

First-line treatment was defined as treatment commenced within six months of initial surgery to account for repeat surgery and delays due to complications. Survival outcomes were calculated from the date of surgery and estimated using the Kaplan–Meier method. Relevant intergroup statistics were used to identify significant differences between groups. Univariate and multivariate analyses were conducted to identify factors influencing treatment choices using variables known to impact prognosis, including gender, age at diagnosis, ECOG status, extent of resection according to either surgeon estimation or post-operative imaging, histological sub-type and risk category. Treatment practices were also compared according to two time periods: January 2016–December 2019 (TP1) and January 2020–December 2022 (TP2).

All analyses were performed using R programming v3.5.1.

## 3. Results

### 3.1. Patient Characteristics

From 1 January 2016 to 31 December 2022 inclusive, 224 patients with grade 2 glioma were identified in the BRAIN registry. Fifty-five percent of patients were male, with approximately 40% being 40 years and older. A majority (72%) of patients had an ECOG performance status of 0–1. The most common tumour type was astrocytoma, with the majority of tumours identified as IDH mutant on immunohistochemistry (see Table 1). Almost 40% were oligodendroglioma. The most common location was the frontal lobe (63%), followed by the parietal lobe (22%), with 8% located in deep structures and 5% being multifocal.

Risk categories as per the definition used in the RTOG 9802 trial [4] were applied. Overall, 83% of patients were deemed high-risk, with 39% of these patients aged less than 40 years with incomplete resection, 47% aged 40 years and over with incomplete resection, and 11% aged 40 years and over with complete resection.

### 3.2. Patient Treatment Practices over Time

**Figure 1:**
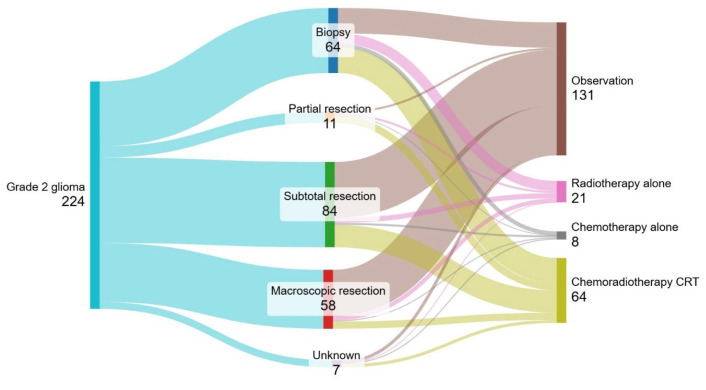
Sankey diagram of patient treatment flows in the first-line setting for all patients.

Figure 1 demonstrates the treatment practices for all patients with grade 2 glioma, allocating patients according to their extent of resection followed by type of adjuvant therapy. Almost 30% (*n* = 64) of patients underwent biopsy only, and 24% (*n* = 58) underwent macroscopic resection. Following surgery, 55% of patients were observed. Characteristics including gender, age, ECOG performance status, extent of resection, risk category and histology were assessed for association with the decision to observe rather than treat after initial surgery. Only macroscopic resection (compared with incomplete resection: 79% vs. 51%, *p* = 0.008) and age less than 40 years (compared with age 40 years and over 78% vs. 39%, *p* < 0.001) were independently associated with observation. Following surgery, 64 patients (27%) received sequential chemotherapy and radiotherapy (CRT). Of those, three patients received multi-agent PCV chemotherapy (consisting of 2 astrocytomas and 1 oligodendroglioma), and 61 patients received single-agent temozolomide.

**Figure 2:**
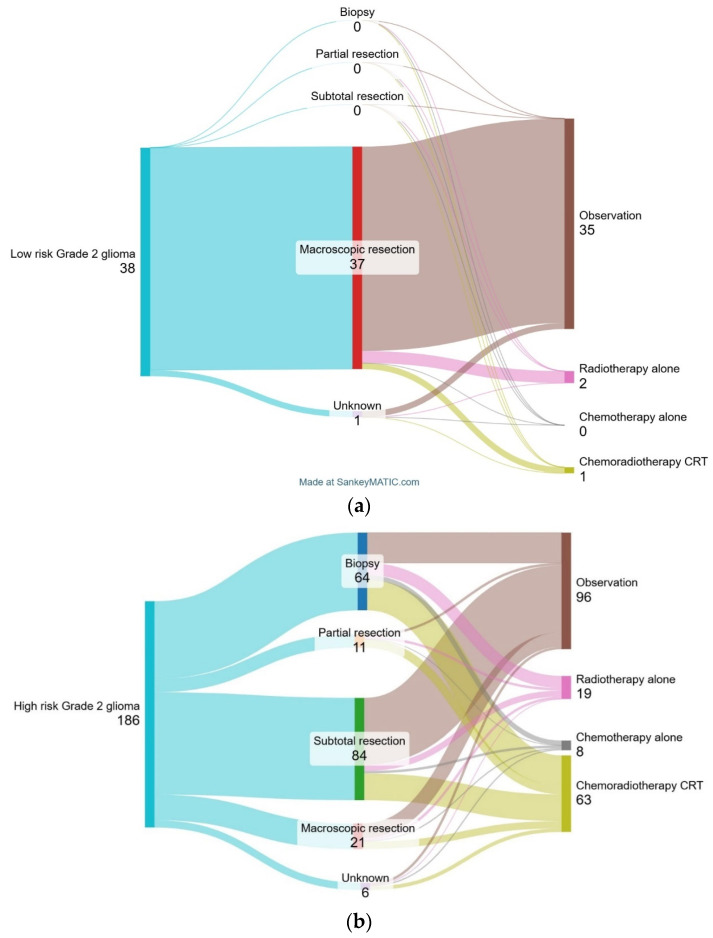
Sankey diagram of patient treatment flows in the first-line setting for all patients with low-risk grade 2 glioma (**a**) and high-risk grade 2 glioma (**b**).

Figure 2 shows treatment practice according to risk category. In the 38 patients (17%) deemed low-risk, 35 patients (91%) underwent observation following resection, 1 patient (3%) received CRT, and 2 patients (6%) received radiotherapy alone (RT).

Of the 186 patients (83%) deemed high-risk, 96 patients (52%) underwent observation following surgery, 63 patients (34%) received CRT, 19 patients (10%) received radiotherapy alone and 8 patients (4%) received chemotherapy alone. Characteristics including gender, age, ECOG PS, extent of resection, risk category and histology were assessed for association with CRT. Compared to low-risk patients, high-risk patients were more likely to have undergone a biopsy (34% vs. 0%; *p* < 0.001) and received to have CRT (34% vs. 3%; *p* < 0.001). High-risk patients who were aged 40 years and older (observation vs. CRT: 45% vs. 76%, *p* < 0.001) were more likely to have received CRT. 

There was no difference in treatment practices based on histology, with approximately 30% of patients with oligodendroglioma, IDH-mutant astrocytoma and IDH-wildtype astrocytoma, respectively, receiving CRT.

Patients were compared across two different time periods: TP1, defined as 2016–2019, and TP2, defined as 2020–2022. There were no significant differences in the median age or gender of patients diagnosed between the two time periods. Over time, there were numerically greater patients with an IDH mutation detected, with the majority detected on immunohistochemistry; however, it did not reach significance (TP1 vs. TP2: 70% vs. 88%, *p* = 0.05). The proportion of high-risk patients remained similar over time (TP1 vs. TP2: 86% vs. 79%, *p* = 0.18). With respect to treatment practice, compared to TP1, patients diagnosed in TP2 were less likely to have undergone biopsy (TP1 vs. TP2: 35% vs. 20%, *p* = 0.02) or received radiotherapy alone (TP1 vs. TP2: 14% vs. 4%, *p* = 0.01) and more likely to have received CRT (TP1 vs. TP2: 22% vs. 36%, *p* = 0.004). The proportion of patients observed was similar (TP1 vs. TP2: 57% vs. 44%, *p* = 0.07).

### 3.3. Subsequent Intervention

At the time of data review, 104 patients (46%) had radiologically progressed at a median follow-up of 35.8 months (range 0.1–98 months). Of these, 69 patients (66%) had been observed post-surgery, and 35 (44%) had received post-surgery anticancer treatment. Subsequent interventions are outlined in Table 2. Of the patients who were observed post-surgery, almost half received chemoradiotherapy, and 29% underwent re-resection. In those who received CRT after initial surgery, twenty patients (31%) had recurred, and of these, only 9 (45%) received treatment at recurrence. Apart from initial treatment received, the only other factor associated with active treatment at recurrence was younger age (age group <40 yrs vs. ≥40 yrs: 87.5% vs. 25%, *p* < 0.001).

### 3.4. Progression-Free and Overall Survival

At a median follow-up of 35.8 months (range 0.1–98 months), 104 (46%) patients had progressed, with a median progression-free survival (PFS) of 47.2 months (95% CI 41.0–53.8). Only 34 (15%) patients died, meaning that the overall survival data is not yet mature. 

**Figure 3:**
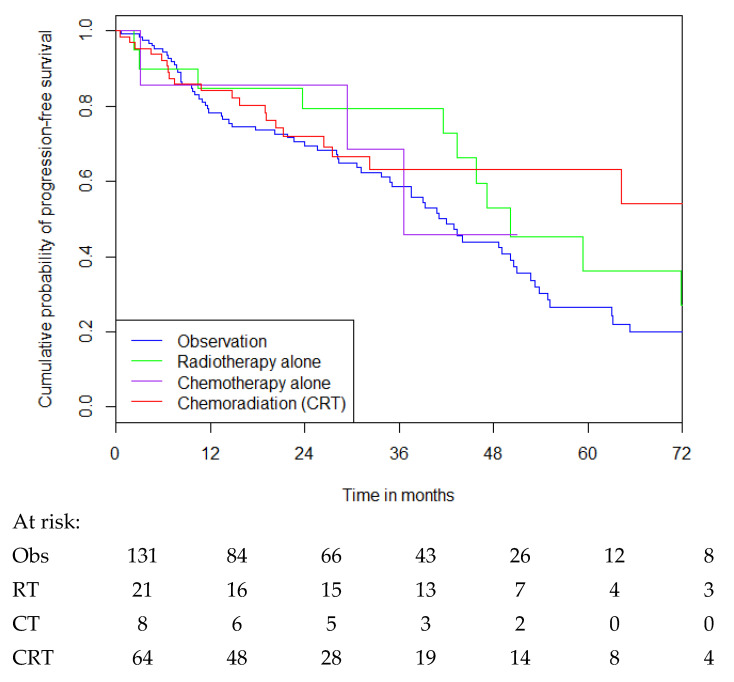
PFS for patients with grade 2 glioma based on treatment received following surgical resection.

Median PFS in all patients, regardless of risk category, who received observation vs. radiotherapy alone vs. chemotherapy alone vs. CRT was 42.0 months vs. 50.2 months vs. 36.6 months vs. not reached, respectively (see Figure 3). CRT was associated with a significant improvement in median PFS over observation alone (HR for disease progression, 0.58 [(0.35–0.95)], *p* = 0.03 by the log-rank test), but not compared with radiotherapy alone (HR 0.8 [(0.38–1.68)], *p* = 0.55), noting small numbers. Of those who underwent observation following surgery, approximately 84 patients (64%) were progression-free at 12 months. 

In high-risk patients, CRT was associated with a significant improvement in median PFS over observation (observation vs. CRT: 39.0 months vs. not reached, HR 0.49 [(0.29–0.83)], *p* = 0.007), but not compared with radiation (radiation vs. CRT: 50.2 months vs. not reached, HR 0.8 [(0.37–1.74)], *p* = 0.57), noting small numbers. Of the 96 (52%) patients who underwent observation, 59 (61%) of these remained progression-free at 12 months and 10 (10%) at 5 years following surgery.

In low-risk patients, over 90% were observed, precluding any analysis of outcome based on treatment received. 

When incorporating IDH status, there was a trend towards worsened median PFS in IDH-wildtype tumours. However, this was not statistically significant (oligodendroglioma vs. IDH-mutant astrocytoma vs. IDH-wildtype astrocytoma: 53.8 months vs. 42.9 months vs. 24.0 months; hazard ratio for disease progression, 1.21 [(0.98–1.49)]; *p* = 0.07).

When comparing risk categories, there was no difference in median PFS between low-risk and high-risk patients irrespective of treatment (low-risk vs. high-risk: 43.4 months vs. 48.6 months; hazard ratio for disease progression, 1.19 [(0.67–2.09)]; *p* = 0.5), nor in those that underwent observation (low-risk vs. high-risk: 43.4 months vs. 39.0 months; hazard ratio for disease progression, 1.67 [(0.89–3.12)]; *p* = 0.1). Similarly, when IDH-wildtype astrocytoma was excluded, there was no difference in median PFS between low-risk and high-risk patients irrespective of treatment (low-risk vs. high-risk: 40.8 months vs. 50.6 months; hazard ratio for disease progression, 1.38 [(0.75–2.54)]; *p* = 0.3), nor in those that underwent observation (low-risk vs. high-risk: 40.8 months vs. 50.2 months; hazard ratio for disease progression, 1.32 [(0.68–2.58)]; *p* = 0.4).

## 4. Discussion

This retrospective review explores the evolution of patterns of care in grade 2 glioma following the publication of a randomised controlled trial that was expected to be practice-changing. Analyses of real-world populations, such as patients entered into the multi-institutional BRAIN registry, provide insight into practice patterns and uptake of a new standard of care. The uptake of sequential chemotherapy and radiation after surgery for high-risk grade 2 glioma is increasing over time. However, overall use remains low, with less than half of high-risk patients receiving CRT. Notably, we saw no difference in outcomes for patients based on their risk category, even when removing IDH-wildtype patients, highlighting the importance of additional research to further validate the definitions of low and high-risk grade 2 glioma. The ability to risk stratify is particularly relevant in the era of IDH inhibitors, which provide an active treatment option for patients with residual disease post-surgery or at the time of recurrence who do not receive chemotherapy or radiotherapy. 

Despite being considered a standard practice since 2016 and endorsed in treatment guidelines, only a minority of high-risk patients received chemoradiation after surgery in our registry. There was also a trend towards increased extent of resection over time, which may be in response to risk stratification for adjuvant therapy selection, evidence with regards to benefit from more extensive resection or increased use of advanced surgical techniques to guide resection such as intraoperative MRI, mapping and 5-aminolevulinic acid, and a decrease in radiation alone, likely due to a preference for sequential treatment. Ultimately, when compared with patients who are observed, early CRT was associated with significantly longer PFS, and the magnitude of this benefit was consistent with the differences observed in the RTOG 9802 study [4]. Whilst our data did not demonstrate a PFS benefit with CRT versus radiation alone, this may be in part due to the modest numbers of patients receiving radiation alone as well as the relatively short follow-up period. There are few studies addressing patterns of care in low-grade glioma over time. The two most notable publications [6,7] both arose from Swedish populations and demonstrated similar findings to our study; however, they analysed patients with both grade 2 and grade 3 gliomas as a single group. Given the differences in biology and treatment between grade 2 and grade 3 gliomas, our study uniquely demonstrates the uptake of sequential therapy in the post-RTOG 9802 era and is an important understanding as we move into the IDH-inhibitor era.

With respect to risk stratification, in 1998, the Radiation Therapy Oncology Group (RTOG) dichotomised low-grade glioma into two distinct groups and embedded two studies within Protocol 9802. This was based on the observed prognostic impact of age and neurosurgeon-determined maximal resection that emerged from prospective clinical trials [3,8], although other groups have used different definitions for high-risk, incorporating tumours crossing the midline and presence of neurological symptoms [9]. In RTOG 9802, patients aged 18–39 years with neurosurgeon-defined macroscopic resection were defined as low-risk and were enrolled in an observational study [9], whilst those aged 40 years and older or any patient with incomplete resection were defined as high-risk and were enrolled in the phase III trial comparing radiotherapy alone or radiotherapy followed by PCV chemotherapy [4]. When comparing the two risk groups, irrespective of the treatment arm in the high-risk patients, overall survival was significantly better in low-risk patients (5-yr OS: 93% vs. 66%, *p* < 0.0001), whereas progression-free survival was similar (5-yr PFS: 48% vs. 50%, *p* = 0.13) [10]. Our study demonstrates similar findings with no significant difference between low-risk and high-risk patients with regard to PFS, including those who undergo observation alone, suggesting that the defined risk categories may not accurately predict PFS. 

This highlights the challenges of differentiating favourable and unfavourable subtypes with respect to PFS, which has become particularly important in the context of the more recent Indigo trial [11]. This phase III double-blind, placebo-controlled trial randomised patients with recurrent or residual grade 2 glioma to either vorasidenib, an IDH inhibitor, or observation alone. The trial met the primary endpoint, demonstrating improved progression-free survival with vorasidenib (median PFS: 27.7 months vs. 11.1 months; HR for disease progression or death, 0.39 [(0.27–0.56)], *p* < 0.001) [10]. However, key inclusion criteria for the trial included residual disease post-surgery and surgery having occurred at least 12 months prior to enrolment. Given all patients with residual disease would be deemed high-risk according to the RTOG study [4], this raises the crucial question of which high-risk patients can be safely observed for 12 months without adjuvant chemoradiation and which requires immediate adjuvant therapy. In our study, 61% of high-risk patients who underwent observation following surgery remained progression-free at 12 months, with 10% remaining progression-free at 5 years, demonstrating a population of high-risk patients achieving clinically meaningful progression-free survival outcomes with surgery alone. Further research is required to identify relevant prognostic biomarkers beyond IDH mutation status that may be included in risk stratification. 

In 2021, the new classification guidelines for glioma highlighted certain molecular markers within both the IDH mutant and IDH wildtype population of grade 2 glioma that predict adverse survival [12]. In our study, we observed an increasing proportion of IDH mutant tumours over time. This is likely in part a result of the increasing use of IDH immunohistochemistry and the availability of next-generation sequencing to identify non-canonical IDH mutations, although less than 10% had sequencing results available. We also noted a trend towards worsened PFS in patients who did not have an IDH mutation. However, we acknowledge that the majority of these patients did not undergo sequencing, and this likely remains a heterogeneous group. Exploration of the impact of therapy based on various IDH mutations was outside the scope of our study. However, further study of differences in molecular status and how these different molecular profiles influence survival or treatment selection should be pursued. 

Limitations exist for this study. Firstly, this is an unplanned retrospective review of data contained within a large prospective registry. Data on decision-making was not collected, and motivation for treatment selection in individual patients, therefore, remains unclear. Secondly, the toxicity associated with therapy, both acute and long-term, is not well captured, and as such, we cannot comment on the impact of any treatment on the lived experience of patients or their quality of life over time. Thirdly, follow-up remains relatively short, which limits the ability to assess and fully appreciate the impact of therapy selection on survival outcomes. Further analysis over time should be pursued as the data mature. Thirdly, the molecular-based classification system was not recognised until June 2021, and as such, the majority of patients in this study did not have additional molecular tests to assess for CDKN2A/B homozygous deletion, EGFR amplification, TERT promoter mutation or chromosome 7 gain or 10 loss. As such, it is unknown whether any of the cases are molecular grade 4 glioma. Finally, the decision to incorporate patients without a known IDH mutation highlights the challenges of implementing molecular-based classification systems in real-world practice. Whilst we acknowledge this creates a heterogeneous group for retrospective analyses such as these, it provides for a more accurate representation of patterns of care in the real world where decisions may be made in the absence of such data. The strength of our study is that it reflects real-world outcomes and the uptake rates of treatment options found to be efficacious in large, randomised studies [3,4].

## 5. Conclusions

In summary, the selection and timing of treatment following surgery in grade 2 glioma is complex and multi-factorial. Whilst CRT is associated with an improved PFS in high-risk grade 2 glioma over observation alone, there has been limited uptake of this approach in the real-world setting. The majority of high-risk patients continue to be observed following initial surgery, especially those that are younger than 40 years or undergo resection rather than biopsy, and a population of these high-risk patients achieve clinically meaningful progression-free survival. Identification of more prognostic biomarkers will assist in determining which patients require immediate treatment and which can be safely observed. 

## Figures and Tables

**Table 1:** Patient baseline characteristics and initial treatment.

Characteristic	Grade 2 Glioma		
	All	High Risk	Low Risk
	*n* = 224	*n* = 186	*n* = 38
Gender			
Male	130 (55%)	105 (56%)	25 (66%)
Female	94 (45%)	81 (44%)	13 (34%)
Age			
Median age (range)	40 years (20–85)	43 years (20–85)	31 years (20–39)
18–29 years	45 (19%)	30 (16%)	15 (39%)
30–39 years	66 (28%)	43 (23%)	23 (61%)
40–64 years	91 (38%)	91 (49%)	0 (0%)
Over 65 years	22 (9%)	22 (12%)	0 (0%)
ECOG			
0	142 (60%)	114 (61%)	28 (74%)
1	29 (12%)	25 (13%)	4 (11%)
2	9 (4%)	9 (5%)	0 (0%)
3+	5 (2%)	5 (3%)	0 (0%)
Missing	39 (16%)	33 (18%)	6 (16%)
Tumour location			
Frontal	150 (63%)	118 (63%)	26 68%)
Parietal	53 (22%)	35 (19%)	4 (10%)
Occipital	9 (4%)	12 (6%)	0 (0%)
Temporal	50 (21%)	53 (28%)	8 (21%)
Deep structures	20 (8%)	18 (10%)	1 (3%)
Multifocal	11 (5%)	11 (6%)	0 (0%)
Histology			
Astrocytoma: IDH mutant	92 (39%)	70 (38%)	22 (58%)
Astrocytoma: IDH wild type	36 (15%)	33 (18%)	3 (8%)
Astrocytoma: NOS	2 (<1%)	1 (<1%)	1 (3%)
Oligodendroglioma	94 (39%)	82 (44%)	12 (32%)
Extent of resection			
Biopsy	64 (27%)	64 (34%)	0 (0%)
Partial resection	11 (5%)	11 (6%)	0 (0%)
Subtotal resection	84 (35%)	84 (45%)	0 (0%)
Macroscopic resection	58 (24%)	21 (11%)	38 (100%)
Missing	7 (3%)	6 (3%)	0 (0%)
Post-operative treatment			
Observation	131 (55%)	96 (52%)	35 (91%)
Radiotherapy alone	21 (9%)	19 (10%)	2 (6%)
Chemotherapy alone	8 (3%)	8 (4%)	0 (0%)
Sequential chemotherapy and radiotherapy	64 (27%)	63 (34%)	1 (3%)

**Table 2:** Practice for recurrent grade 2 glioma.

Characteristic	Relapsed Grade 2 Glioma (*n* = 104)			
	Observed	RT	Chemotherapy	CRT
	*n* = 69	*n* = 12	*n* = 3	*n* = 20
Best supportive care	5 (7%)	2 (17%)	0 (0%)	11 (55%)
Surgery alone	20 (29%)	2 (17%)	0 (0%)	5 (25%)
RT	4 (6%)	0 (0%)	1 (33%)	0 (0%)
Chemotherapy	7 (10%)	7 (58%)	0 (0%)	4 (20%)
CRT	33 (48%)	1 (8%)	2 (67%)	0 (0%)

## Data Availability

The data used in this study are not publicly available as this would compromise study ethics; however, it may be made available upon reasonable request to the corresponding author.
